# Multidimensional Analyses of Tumor Immune Microenvironment Reveal the Possible Rationality of Immunotherapy and Identify High Immunotherapy Response Subtypes for Renal Papillary Cell Carcinoma

**DOI:** 10.3389/fimmu.2021.657951

**Published:** 2021-08-31

**Authors:** Baojun Wei, Meng Yu, Jihang Yao, Mingzhe Jiang, Jun An, Jieping Yang, Jiaxing Lin, Yongkang Zhao, Yuyan Zhu

**Affiliations:** ^1^Department of Urology, The First Hospital of China Medical University, Shenyang, China; ^2^Department of Laboratory Animal Science, China Medical University, Shenyang, China; ^3^Key Laboratory of Transgenic Animal Research, China Medical University, Shenyang, China; ^4^Department of Gynecology, The First Hospital of China Medical University, Shenyang, China; ^5^National Institute of Health and Medical Big Data, China Medical University, Shenyang, China; ^6^Joint Laboratory of Artificial Intelligence and Precision Medicine of China Medical University and Northeastern University, Northeastern University, Shenyang, China

**Keywords:** renal papillary cell carcinoma, CD8+ T-cell exhaustion, *CCL5*, *FASLG*, immunotherapy response subtypes

## Abstract

Kidney renal papillary cell carcinoma (KIRP), the second most common subtype of renal cell carcinoma, still lacks effective treatment regimens for individualized immunotherapy because of the heterogeneity of its elusive immune microenvironment. Therefore, we aimed to comprehensively evaluate the immune microenvironment of KIRP by using the computational biology strategy to analyze the expression profile data of 289 KIRP patients obtained from The Cancer Genome Atlas database. Based on multidimensional, multi-omics bioinformatics analysis, we found that the tumor of patients with KIRP exhibited “hot” tumor characteristics but the CD8+ T cells in the tumor tissues did not limit tumor progression. Thus, patients with KIRP may realize higher clinical benefits by receiving treatment that can reverse CD8+ T-cell exhaustion. Among them, C1 and C3 immune subtypes could realize the best efficacy of reversing CD8+ T-cell exhaustion. Moreover, *CCL5* and *FASLG* expression may be related to the formation of the immunosuppressive microenvironment in the tumors of patients with KIRP. In conclusion, the immune microenvironment landscape presented in this study provides a novel insight for further experimental and clinical exploration of tailored immunotherapy for patients with KIRP.

## Introduction

Renal cell carcinoma (RCC) is one of the most common types of kidney cancer in humans. In the past two decades, the incidence of kidney cancer has been increasing (accounting for 2%–3% of all new tumor cases) and RCC accounting for approximately 85% of kidney cancer cases (1). Kidney renal papillary cell carcinoma (KIRP) is the second most common type of RCC, accounting for 10%–20% of all RCC cases (2). Although considerable progress has been made in the diagnosis and treatment of RCC, the clinical outcome of RCC is still not satisfactory (3, 4). As an important subtype of kidney cancer, the preferred treatment for local and locally advanced KIRP is surgical resection. Although multiple drugs have been used for treating advanced and metastatic KIRP, including anti-vascular endothelial factor drugs and mTOR inhibitors, effective and individualized immunotherapeutic programs for treating KIRP have still not been developed (2).

Tumor-infiltrating immune cells (TIICs) form an ecosystem that regulates cancer progression in the tumor microenvironment and have exhibited potential prognostic value (5). The most studied TIICs are tumor-infiltrating lymphocytes, among which cytotoxic CD8+ T cells can prevent tumor growth (6). However, antigen-epitope-specific CD8+ T cells present in the tumor *in situ* or the peripheral blood are often inactivated first (7, 8). The combination of the *CTLA4–B7* complex on the surface of T cells inhibits the activation of T cells, leading to the inhibition of T-cell proliferation (9). A previous study has shown that *TIM-3* and *PD-1* have a potential synergistic effect and induce the dysfunction of tumor-infiltrating CD8+ T cells (10). Therefore, these lead to the exhaustion of CD8+ T cells in the tumor microenvironment and, thus, the loss of antitumor effects. At present, targeted drugs for *PD-1* and *CTLA4* have been approved treating various cancers including KIRC and have achieved good results (11–15). The size and number of intraperitoneal and retroperitoneal metastases were considerably reduced in a patient with type 2 recurrent metastatic KIRP after 5 months of treatment with nivolumab monoclonal antibody (16), suggesting the potential feasibility of restoring the antitumor ability of CD8+ T cells to treat KIRP. However, a comprehensive evaluation of the tumor immune microenvironment in patients with KIRP has still not been performed, which has severely limited the further exploration of the feasibility of immunotherapy in these patients.

Therefore, in this study, we comprehensively evaluated the tumor immune microenvironment of patients with KIRP and identified key genes and T-cell subsets that are closely related to the tumor microenvironment of patients with KIRP and can be used as immunotherapeutic targets or markers. Furthermore, we identified subtypes with different immunotherapy responses of KIRP patients through an unsupervised clustering method and further demonstrated the prospect of immunotherapy based on reversing CD8+ T-cell exhaustion in KIRP patients.

## Materials and Methods

### Data Sources and Differential Analysis of mRNAs

The RNA-seq matrix files (count format) and clinical information of 289 KIRP samples and 32 peritumoral kidney samples were downloaded from The Cancer Genome Atlas (TCGA) (https://cancergenome.nih.gov/) (**Tables 1** and **2**). The mRNA-seq matrix files were extracted for the next differential analysis. The R package “edgeR” was applied to screen differentially expressed mRNAs between normal and tumor tissues. Next, the p-value was calculated by the FDR-corrected method. The mRNAs with fold change >2 (| log2 fold-change | >1) and p < 0.05 were filtrated as differentially expressed genes.

**Table 1:** Summary of clinical characteristics of histological subtypes in the KIRP-TCGA cohort.

	Histological subtypes		
	Type 1 (N = 76)	Type 2 (N = 82)	NA (N = 131)
Age (year)	58 (28–79)	64 (28–85)	62 (31–88)
Gender			
Male (%)	73.7	69.5	76.3
Female (%)	26.3	30.5	23.7
Stage			
I (n)	57	42	73
II (n)	3	5	13
III (n)	3	24	24
IV (n)	1	6	8
NA (n)	12	5	13
T stage (n) 1/2/3/4/NA	64/7/4/1/0	47/8/26/1/0	82/18/29/0/2
N stage (n) 0/1/2/NA	8/2/0/66	14/13/3/52	28/9/1/93
M stage (n) 0/1/NA	17/0/59	29/3/50	48/6/77

NA, data lost or unknown.

**Table 2:** Summary of clinical characteristics of molecular subtypes in the KIRP-TCGA cohort.

	Molecular subtypes				
	KIRP.C1 (N = 92)	KIRP.C2a (N = 34)	KIRP.C2b (N = 21)	KIRP.C2c–CIMP (N = 9)	NA (N = 133)
Age (year)	59 (38–83)	64 (42–85)	64 (37–83)	45 (28–61)	63 (28–88)
Gender					
Male (%)	79.3	70.6	57.1	33.3	75.9
Female (%)	20.7	29.4	42.9	66.7	24.1
Stage					
I (n)	70	20	4	0	78
II (n)	5	1	0	1	14
III (n)	8	9	14	5	15
IV (n)	1	3	3	3	5
NA (n)	8	1	0	0	21
T stage (n) 1/2/3/4/NA	75/9/7/1/0	21/2/11/0/0	5/0/16/0/0	0/2/7/0/0	92/20/18/1/2
N stage (n) 0/1/2/NA	11/1/0/80	9/4/1/20	5/6/1/9	1/6/1/1	24/7/1/101
M stage (n) 0/1/NA	25/0/67	19/2/13	12/2/7	2/2/5	36/3/94

NA, data lost or unknown.

### Evaluation of Immune Infiltration

The CIBERSORT method is used to evaluate the proportion of immune cells based on the standardized gene expression files. This method has been validated in gene expression file studies measured by gene chips (17). We used the CIBERSORT to calculate the proportion of 22 immune cells (B cells naïve, B cells memory, plasma cells, T cells CD8, T cells CD4 naïve, T cells CD4 memory resting, T cells CD4 memory activated, T cells follicular helper, T cells regulatory (Tregs), T cells gamma delta, NK cells resting, NK cells activated, monocytes, macrophages M0, macrophages M1, macrophages M2, dendritic cells resting, dendritic cells activated, mast cells resting, mast cells activated, eosinophils, and neutrophils). Samples with p < 0.05 indicated that the proportion of immune cells calculated by CIBERSORT was correct. In addition to CIBERSORT, we used the tumor immune estimation resource (TIMER) database (https://cistrome.shinyapps.io/timer/) to assess the abundance of six immune cells (B cells, CD4+ T cells, CD8+ T cells, neutrophils, macrophages, and myeloid dendritic cells) for the TCGA and GEO cohorts (18).

### Construction of Weighted Co-Expression Network

The regulation network method is widely applied to analyze the gene expression data. When compared with the node-based method, the regulation network method pays attention to both the difference in and the correlation between the gene expression profiles (19). The weighted gene co-expression network analysis (WGCNA) was superior to several other methods in constructing the co-expression networks (20). To determine the gene groups with similar expression patterns, we applied the previously reported R package “WGCNA” to construct a weighted co-expression network for the TCGA cohort (21). First, the outlier samples were filtered to reduce the differences. The soft threshold method was selected to construct the correlation network so that the adjacency matrix becomes a continuous value between 0 and 1. The network thus constructed was closer to the real biological network state. Then, the BlockwiseModules function was applied to build scale-free networks, after which the module division analysis and dynamic tree-cutting algorithm were applied to group the genes with similar expression patterns and define the modules (22). In order to obtain a large number of genes for each module, we selected a dividing line (0.25) to merge some of the modules with a high correlation.

### Identification of Important Modules Related to Immune Cells

To determine the important modules related to T cells in the TCGA cohort, we calculated the value of module eigengenes (MEs), gene significance (GS), and module significance (MS). MEs are the main components of gene principal component analysis in the module. By calculating the correlation between MEs and clinical information, vital relevant modules could be determined. GS is a log10 transformation of p-value in the linear regression between the gene expression and clinical information, which represents the correlation between genes and samples. MS is the average GS in the module. The module with the highest correlation with the CD8+ T cells was selected as the module to be analyzed. The enrichment pathways of the key modules were obtained using the Metascape (http://metascape.org/gp/index.html).

### Construction of the PPI Network and Screening of Its Hub Genes

For the TCGA cohort, the protein–protein interaction (PPI) network in the hub module was constructed using default parameters of the STRING database (23) and then visualized using Cytoscape. The hub genes in the network were calculated using the MCC algorithm in CytoHubba. When compared with the 10 other previously reported methods, MCC can more accurately identify the central objects in the network (24).

### Screening of Hub Genes in the WGCNA Network

For the TCGA cohort, the node with weights >0.4 between the two nodes in the key module was input into the Cytoscape, after which the hub genes in the co-expression network were calculated using the MCC algorithm of CytoHubba. The genes highly correlated with certain clinical features in the modules were identified as the more important genes in the module; therefore, we defined genes that simultaneously satisfied the module membership (MM) >0.9 and gene significance (GS) >0.6 as the hub genes in the WGCNA network.

### Identification of Real Hub Genes in the Key Module

We identified the genes obtained from the intersection of hub genes of the PPI network and the WGCNA network as the hub genes of the key module in the TCGA cohort. The selection of the intersection and drawing of the Wayne diagram was performed using the online tool VENNY (Https://bioinfogp.cnb.csic.es/tools/venny/index.html) (25). After obtaining the true hub genes, we used the R package “corrplot” to draw the correlation heat map between these hub genes.

### Immunohistochemistry and Real-Time Quantitative PCR Analyses

In total, 30 KIRP tissues and 10 adjacent normal tissues were collected from 30 patients who underwent partial nephrectomies or nephrectomies at The First Hospital of China Medical University. All patients provided their written informed consent. Total RNA was extracted using the TRIzol reagent (Waltham, Massachusetts, USA). The PrimeScript RT Kit (Takara Bio, Inc., Dalian, China) was used to reversely transcribe RNA into cDNA in accordance with the manufacturer’s protocols. The SYBR Green PCR Kit (Takara Bio, Inc., Dalian, China) was used to conduct real-time fluorescence quantitative PCR with the ABI7500 Fluorescent Quantitative PCR Machine (Applied Biosystems, Lincoln Centre Drive Foster City, CA, USA). Glyceraldehyde 3-phosphate dehydrogenase (GAPDH) was used as the internal control. The primer sequences in this experiment were as follows: CCL5 (forward: 5′-CCAGCAGTCGTCTTTGTCAC-3′; reverse: 5′-CTCTGGGTTGGCACACACTT-3′), FASLG (forward: 5′-TGCCTTGGTAGGATTGGGC-3′; reverse: 5′-GCTGGTAGACTCTCGGAGTTC-3′), EOMES (forward: 5′-GCCATGCTTAGTGACACCGA-3′; reverse: 5′-GGACTGGAGGTAGTACCGC-3′), PDCD1 (forward: 5′-CCAGGATGGTTCTTAGACTCCC-3′; reverse: 5′-TTTAGCACGAAGCTCTCCGAT-3′), and GAPDH (forward: 5′-ACAACTTTGGTATCGTGGAAGG-3′ reverse: 5′-GCCATCACGCCACAGTTTC-3′). The mRNA expression levels were analyzed using the 2^-ΔΔCT^ method. The immunohistochemical data of the hub genes were obtained from The Human Protein Atlas (HPA) database (http://www.proteinatlas.org) (26). The method of the immunohistochemical analysis of the HPA could be found in the HPA website (https://www.proteinatlas.org/about/assays+annotation#ih).

### Overall Survival Analysis of KIRP Patients in the TCGA Cohort

Survival-related hub genes were determined by the Kaplan–Meier (KM) survival analysis on the UALCAN (http://ualcan.path.uab.edu/analysis.html) (27). KIRP patients were categorized into the high expression (with gene expression levels above the upper quartile) group and the low/medium expression (with gene expression levels below the upper quartile) group. The log-rank p < 0.05 was considered to be statistically significant.

### Correlation Analysis of Hub Genes and Immune Cells

TIMER (https://cistrome.shinyapps.io/timer/) was used for correlational analysis between the hub genes and immune cells in the TCGA cohort. The correlation analysis between the immune cells and the clinical prognosis was also performed on the TIMER. The R software was used to draw box plots of the proportion of CD8+ T cells and Treg cells in different clinical stages.

### Identification of the Immune Subtypes by Consensus Clustering

We used the R package “ConsensusClusterPlus” to perform consensus clustering and the screening of the immune subtypes of KIRP in the TCGA cohort based on the 12 hub genes of the CD8+ T-cell-related module. In order to present the immune landscapes in different immune subtypes, we used the R package “ESTIMATE” to calculate the immune score, stromal score, and tumor purity of each tumor sample. The package “GSVA” was used to evaluate the single-sample gene-set enrichment analysis (ssGSEA) score based on 29 immune gene sets. The Kaplan–Meier (KM) survival curves of the immune subtypes were performed by using the R package “Survival.”

### Prediction of Immunotherapy Responses in KIRP Patients

“TIDE” is a computational method that calculates the score of T-cell exclusion and T-cell dysfunction to predict the responses of immunotherapy (28). A lower TIDE score predicted a higher immunotherapy response. We classified KIRP patients into a true response group and a false response group by the median of TIDE scores in the TCGA cohort.

### Acquisition and Processing of Validation Datasets

Three expression matrix files of KIRP, GSE2748 (n = 34), GSE7023 (n = 35), and GSE26574 (n = 34) were extracted from the Gene Expression Omnibus (GEO) (https://www.ncbi.nlm.nih.gov/geo/). After merging the three expression matrix files, the batch effects were adjusted by the R package “sva.” The merged files were then used as the validation cohort.

### Identification of Frequently Mutated Genes and Gene-Set Enrichment Analysis in Different Immunotherapy Response Groups

The somatic mutation files (maf format) of 273 KIRP patients of the TCGA cohort were downloaded from TCGA (https://cancergenome.nih.gov/). The R package “Maftools” was used to draw the waterfall plots in order to visualize the genes with mutation frequency >5% in the high and low/medium immunotherapy response groups. The r package “clusterProfiler” was used to perform GSEA analysis to evaluate the biological mechanisms between the high and low/medium immunotherapy response groups. We visualized the top five enrichment results of the high immunotherapy response group.

### Statistical Analyses

Spearman correlation was applied to calculate the correlation coefficients between the hub genes. Wilcoxon test and Kruskal–Wallis test were applied to separately conduct the group comparisons of two groups and more than two groups. Overall survival curves were generated using the Kaplan–Meier method, and the group comparisons were performed with the log-rank test.

## Results

### Construction of a Weighted Co-Expression Network of Differentially Expressed Genes and Evaluation of Immune Infiltration

Under the condition of | log2 (fold-change) |> 1 and p < 0.05, 5,132 differentially expressed genes were screened (3,130 upregulated and 2,002 downregulated) in the TCGA cohort (**Supplementary Figure 1A**). The 5,132 differential genes in 289 tumor samples were used to construct a co-expression network. The optimal soft threshold β = 5 was used for the next calculation (**Supplementary Figure 1B**). The modules with ME of <0.25 in the cluster were merged, and 32 modules were finally obtained in the TCGA cohort (**Supplementary Figures 1C, D**). In the results of CIBERSORT, 137 tumor samples with a p < 0.05 were selected (**Supplementary Figure 1E**).

### Correlation Between the Purple Module and T Cells

**Figure 1:**
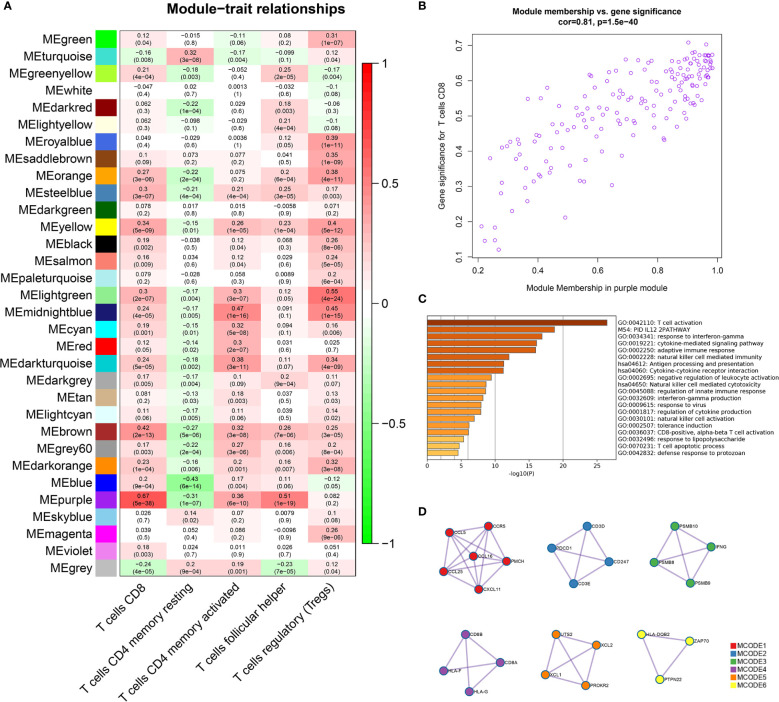
Identification of important modules related to T cells through WGCNA. **(A)** The correlation between the modules and the five T-cell subtypes. The scale bar indicated the range of the correlation coefficient, and the p value is in the parentheses. **(B)** Correlation between purple module members and gene significance. **(C)** GO and KEGG enrichment analysis of 169 genes in the purple module confirmed that these genes were mainly enriched in the process of T-cell activation and antigen processing and presentation. **(D)** Six tightly linked networks in the purple module were obtained using the MCODE algorithm.

In the WGCNA analysis results, we found a high correlation between the purple module (169 genes) and CD8+ T cells (r = 0.67, p = 5e-38) (**Figure 1A**). We also plotted the relationship diagram of GS and MM of the purple module (**Figure 1B**). These results indicate that the purple module was significantly related to CD8+ T cells.

### Analysis of the Function and Pathway of Genes Identified in the Purple Module

The genes of the purple module were related to a variety of immune functions. The first three significantly enriched GO biological processes in the purple module were about T-cell activation (Log10(P) = −26.47), response to interferon-gamma (Log10(P) = −16.90), and the cytokine-mediated signaling pathway (Log10(P) = −16.10). The first three KEGG pathways were antigen processing and presentation (Log10(P) = −11.27), cytokine–cytokine receptor interaction (Log10(P) = −11.19), and natural killer cell-mediated cytotoxicity (Log10(P) = −8.66) (**Figure 1C**, **Supplementary Table 1**). In addition, by using the MCODE algorithm of the Metascape platform, six closely linked network components were calculated and their functionally analyzed (**Figure 1D**, **Supplementary Table 2**).

### Screening of Hub Genes of the Purple Module

**Figure 2:**
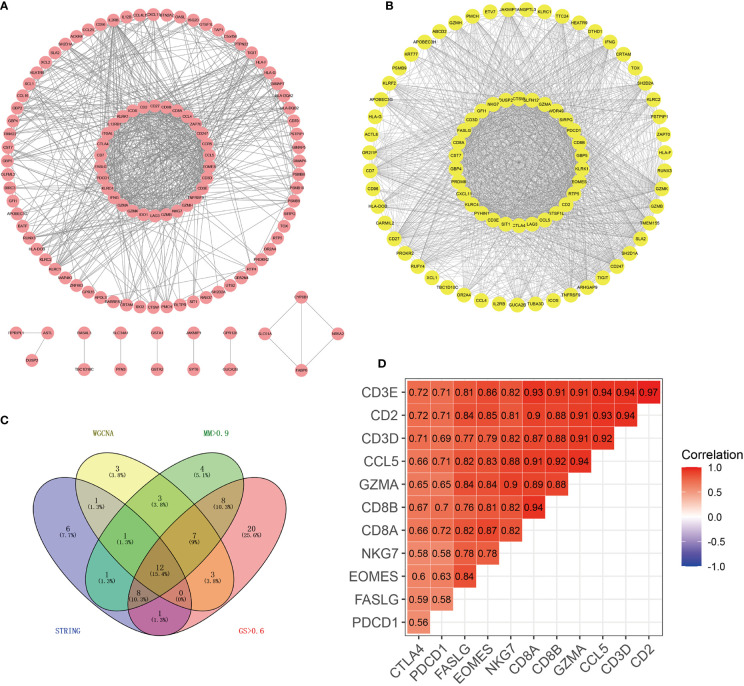
Identification of 12 hub genes by PPI and WGCNA co-expression networks. **(A)** PPI network of genes in the purple module. Using the MCC algorithm, the top 30 genes in the PPI network were the hub genes of the PPI network. **(B)** Weighted co-expression network of nodes with weights > 0.4 between two nodes in the purple module. The top 30 genes in the network scored by the MCC algorithm were the hub genes of the co-expression network. **(C)** The Wayne diagram showed the common hub genes in the PPI network and the co-expression network. **(D)** The correlation heat map showed the correlation coefficients among 12 hub genes.

A PPI network with 115 nodes and 661 edges was generated and visualized, and the genes in the top 30 scores were identified as hub genes of this network (**Figure 2A**). The nodes with a weight of >0.4 (81 nodes, 1,530 edges) between two nodes in the weighted co-expression network were visualized, and the genes in the top 30 scores were identified as hub genes of the weighted co-expression network (**Figure 2B**). In the purple module, genes that were simultaneously satisfied MM >0.9 and GS >0.6 were selected. Then, they were intersected to obtain 12 genes (*GZMA*, *FASLG*, *CD2*, *PDCD1*, *CCL5*, *CD8A*, *CD3D*, *EOMES*, *NKG7*, *CD3E*, *CD8B*, and *CTLA4*) (**Figure 2C**). We believed that these 12 genes were the true hub genes in the purple module and they were strongly correlated (**Figure 2D**).

### Differential Expression of Hub Genes and Survival Analyses

**Figure 3:**
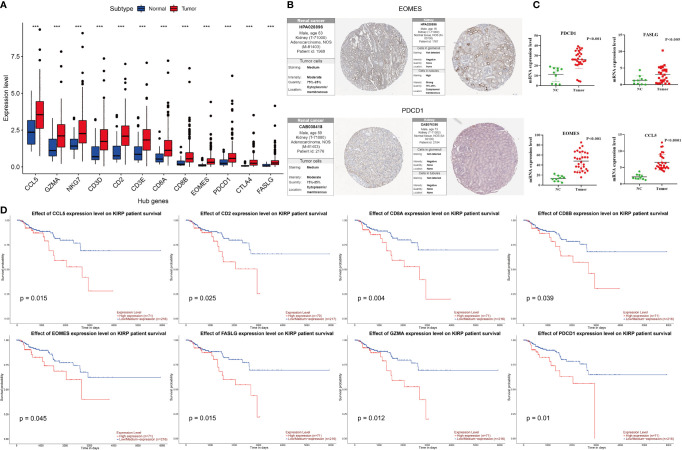
Differential expression of hub genes in KIRP and survival curves of hub genes. **(A)** In the TCGA cohort, the expression of 12 hub genes in the tumor were higher than that in normal tissues, ***p < 0.001. **(B)** Immunohistochemical results of EOMES and PDCD1 in tumor and normal tissues. **(C)** mRNA expression levels of CCL5, FASLG, EOMES, and PDCD1 detected by RT-qPCR in tumor and normal tissues. **(D)** The high expressions (red line) of CCL5, CD2, CD8A, CD8B, EOMES, FASLG, GZMA, and PDCD1 were associated with poor prognosis.

As shown in **Figure 3A**, in the TCGA cohort, the expression of these 12 genes in tumor tissues was higher than that in normal tissues (p <0.05). The immunohistochemical data from HPA showed that *EOMES* and *PDCD1* exhibited medium staining in the cytoplasm and cytomembrane of the tumor cells in the renal cancer. Normal kidney tissue staining of *EOMES* exhibited high staining in the cytoplasm and cytomembrane of cells in tubules, but as for cells in glomeruli, the staining was not detected. The staining of *PDCD1* was not observed in normal kidney tissues (**Figure 3B**). In our samples, the mRNA levels of *CCL5*, *FASLG*, *EOMES*, and *PDCD1* in tumor tissues were significantly higher than those in normal tissues (**Figure 3C**). Survival analysis revealed that the overexpression of eight genes was significantly associated with poor prognosis. As shown in **Figure 3D**, the p values of these genes are as follows: *CCL5* (p = 0.015), *CD2* (p = 0.025), *CD8A* (p = 0.004), *CD8B* (p = 0.039), *EOMES* (p = 0.045), *FASLG* (p = 0.015), *GZMA* (p = 0.012), and *PDCD1* (p = 0.01).

### Hub Genes Were Significantly Correlated With the Fraction of CD8+ T Cells

**Figure 4:**
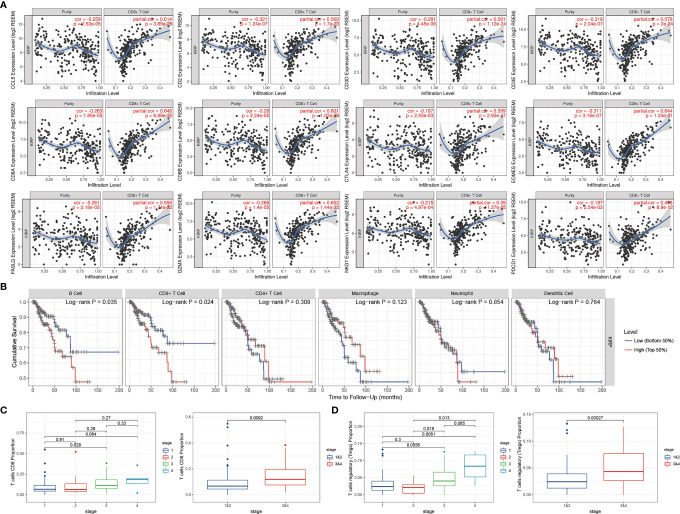
The expressions of hub genes were positively correlated with the infiltration level of CD8+ T cells. **(A)** The expression of 12 hub genes showed a negative correlation with tumor purity, and a positive correlation with the infiltration level of CD8+ T cells. **(B)** Relationship between immune cell infiltration and overall survival in KIRP patients. **(C)** As the tumor progresses, the proportion of CD8+ T cells continues to rise. **(D)** The proportion of Treg cells continues to rise as the tumor progresses.

We validated the significant correlation between hub genes and the fraction of CD8+ T cells in the TCGA cohort by using the TIMER online platform (**Figure 4A**). The high infiltration status of B cells and CD8+ T cells was associated with poor prognosis in patients with KIRP (**Figure 4B**), and the number of CD8+ T cells and Treg cells increased with tumor progression (**Figures 4C, D**).

### Immune Microenvironment Landscapes of the Five Immune Subtypes

**Figure 5:**
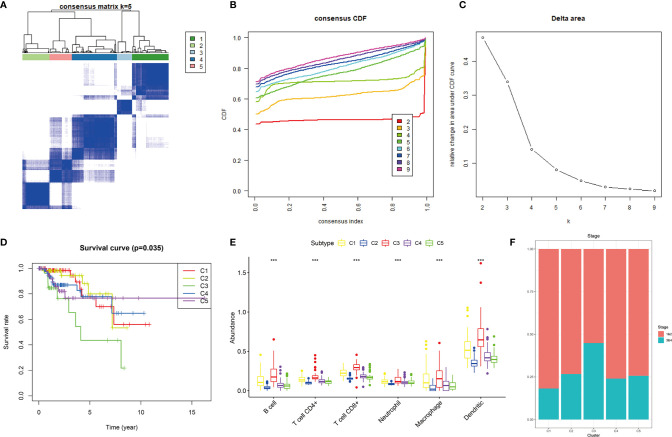
Consensus clustering of KIRP samples in the TCGA cohort. **(A)** Heat map of sample clustering at k = 5. **(B)** Cumulative distribution function CDF curve for k = 2–9. **(C)** Relative change in area under the CDF curve when k = 2–9. **(D)** Survival curve of five immune subtypes. **(E)** The abundance of six immune cells among five immune subtypes, ***p < 0.001. **(F)** Histogram of tumor stages of five immune subtypes.

**Figure 6:**
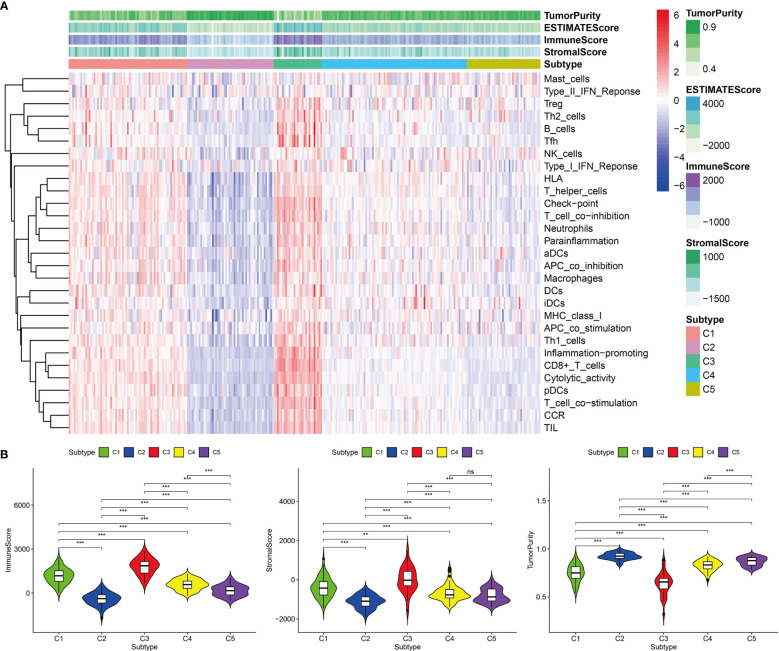
Immune components of five immune subtypes in the TCGA cohort. **(A)** Heat map of 29 immune gene sets of five immune subtypes. **(B)** The immune score, stromal score and tumor purity of five immune subtypes, ***p < 0.001, **p < 0.01, ns p > 0.05.

Based on the 12 hub genes, the KIRP samples of the TCGA cohort were clustered into five subtypes (C1: 71 samples, C2: 52 samples, C3: 29 samples, C4: 87 samples, and C5: 44 samples) (**Figures 5A–C**). The prognostic outcomes of the five subtypes showed significant statistical differences (**Figure 5D**). The abundance of six types of immune cells (B cells, CD4+ T cells, CD8+ T cells, neutrophils, macrophages, and myeloid dendritic cells) was compared among the five subtypes (**Figure 5E**). The number of patients at different tumor stages in the immune subtypes is shown in **Figure 5F**. We also compared the ssGSEA score, immune score, stromal score, and tumor purity of the five immune subtypes (**Figures 6A, B**). Among the immune subtypes, C1 and C3 showed the highest abundance of the six immune cell types with the highest ssGSEA, immune, and stromal scores. C4 and C5 showed a moderate abundance of the six immune cell types along with moderate ssGSEA, immune, and stromal scores. Notably, C2 showed the lowest abundance of the six immune cell types with the lowest ssGSEA, immune, and stromal scores. On the other hand, tumor purity showed the opposite trend among these immune subtypes. Interestingly, in C1 and C3, which were rich in immune components, the percentage of patients at high tumor stages (III/IV) in C1 was 18.2%, and that in C3 was 44.8%.

### Predictions of Immunotherapeutic Responses Among the Five Immune Subtypes

**Figure 7:**
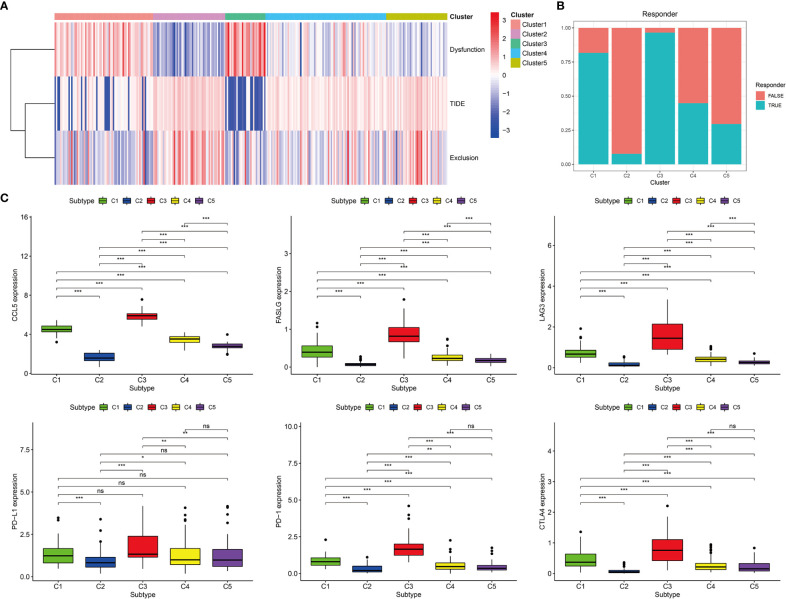
Prediction of immunotherapy response of five immune subtypes in the TCGA cohort. **(A)** Heat map of dysfunction scores, exclusion scores, and TIDE scores of five immune subtypes. **(B)** Histogram of responder of five immune subtypes (true responder: C1 81.7%, C2 7.7%, C3 96.6%, C4 44.8%, C5 29.5%). **(C)** Gene expression levels of PD-1, PD-L1, CTLA4, LAG3, FASLG, and CCL5 among five immune subtypes, ***p < 0.001, **p < 0.01, *p < 0.05, ns p > 0.05.

Among the five immune subtypes, C1 and C3 had the lowest TIDE and highest T-cell dysfunction scores. C4 and C5 had moderate TIDE and T-cell dysfunction scores. C2 showed the highest TIDE and lowest T-cell dysfunction scores (**Figure 7A**). The details of TIDE and T-cell dysfunction scores of each KIRP sample are presented in **Supplementary Table 3**. A sample with the TIDE score greater than 1.15 was defined as a false responder to immunotherapy, and a sample with the TIDE score less than 1.15 was defined as a true responder to immunotherapy. The percentage of true responders to immunotherapy decreased in the following order: C3 (96.6%) > C1 (81.7%) > C4 (44.8%) > C5 (29.5%) > C2 (7.7%) (**Figure 7B**). The expression of four immune checkpoint genes (*LAG3*, *PD-L1*, *PD-1*, and *CTLA4*) and two hub genes (*CCL5* and *FASLG*) decreased in the following order: C3 > C1 > C4 > C5 > C2 (**Figure 7C**). Based on these findings, C1 and C3 were considered the high immunotherapy response group, C4 and C5 were considered the median immunotherapy response group, and C2 was considered the low immunotherapy response group.

### Validation of the Immune Subtypes of KIRP

**Figure 8:**
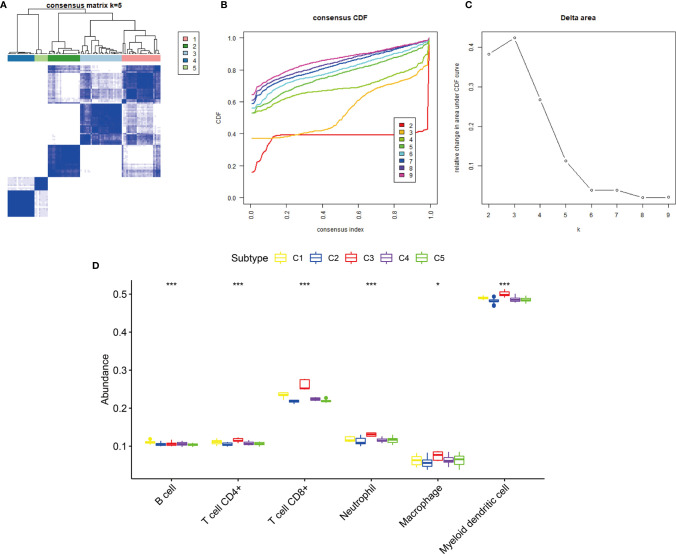
Consensus clustering of KIRP samples in the GEO cohort. **(A)** Heat map of sample clustering at k = 5. **(B)** Cumulative distribution function (CDF) curve for k = 2–9. **(C)** Relative change in area under the CDF curve when k = 2–9. **(D)** The abundance of six immune cells among five immune subtypes, ***p < 0.001, *p > 0.05.

**Figure 9:**
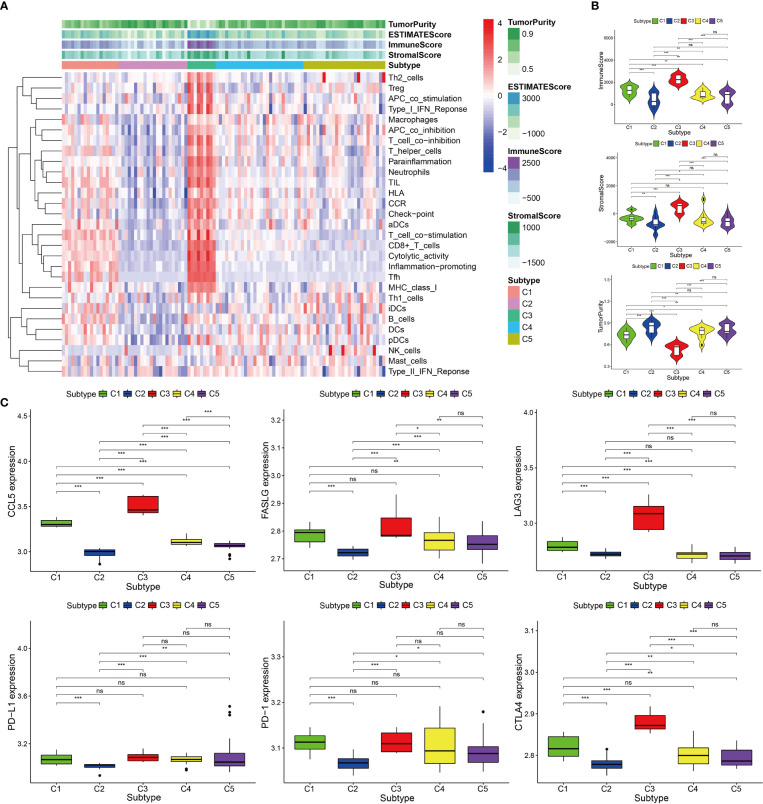
Immune components and expression levels of immune checkpoints among five immune subtypes in the GEO cohort. **(A)** Heat map of 29 immune gene sets of five immune subtypes. **(B)** The immune scores, stromal scores, and tumor purity of five immune subtypes. **(C)** Gene expression levels of PD-1, PD-L1, CTLA4, LAG3, FASLG, and CCL5 among five immune subtypes. ***p < 0.001, **p < 0.01, *p < 0.05, ns p > 0.05.

The GEO cohort was clustered into five subtypes based on the 12 hub genes to validate the immune subtypes of KIRP as identified in the TCGA cohort (**Figures 8A–C**). C1 and C3 had the highest abundance of immune cells, especially CD8+ T cells and myeloid dendritic cells. This observation was consistent with the results of the TCGA cohort (**Figure 8D**). In terms of immune-related gene-set enrichment score, immune score, stromal score, and tumor purity, the analysis results of the GEO cohort showed the same trend as that of the TCGA cohort (**Figures 9A, B**). In terms of the expression of two hub genes (*CCL5* and *FASLG*) and four immune checkpoint genes (*LAG3*, *PD-L1*, *PD-1*, and *CTLA4*), the analysis results of the GEO cohort also exhibited the same trend as that of the TCGA cohort (**Figure 9C**). These comparable results between the TCGA and GEO cohorts indicated that the immune subtypes of KIRP identified in this study existed in KIRP.

### Comparing the Immune Subtypes With Histological and Molecular Subtypes

**Figure 10:**
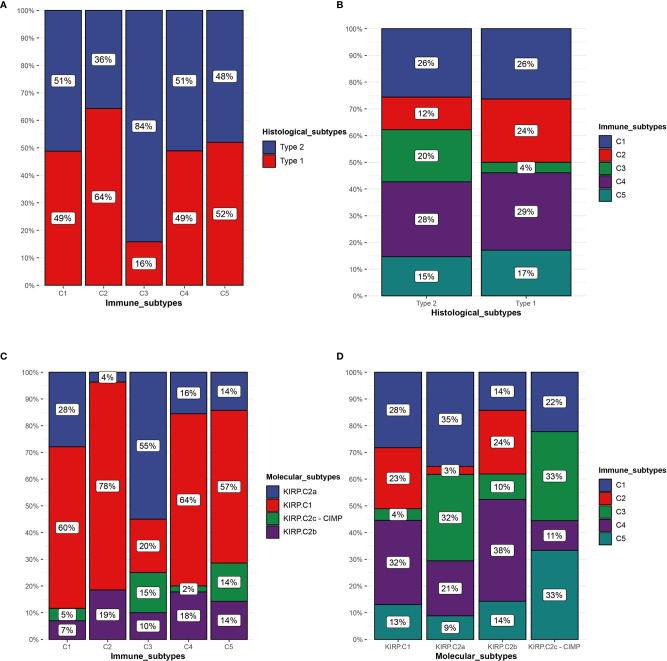
Comparison of multiple KIRP subtypes. **(A)** Percentage of histological subtypes in immune subtypes. **(B)** Percentage of immune subtypes in histological subtypes. **(C)** Percentage of molecular subtypes in immune subtypes. **(D)** Percentage of immune subtypes in molecular subtypes.

Some studies have previously proposed the existence of two different histological subtypes and four different molecular subtypes in KIRP: type 1 with pale cytoplasm and small cells and type 2 with eosinophilic cytoplasm and large cells. Type 2 KIRP presented at a higher tumor stage more often than type 1 (29, 30). Molecular subtype KIRP.C1 was enriched in type 1 KIRP and associated with the *MET* mutation and gain of chromosome 7. Molecular subtype KIRP.C2a was enriched in type 2 KIRP and associated with DNA methylation cluster 2. Molecular subtype KIRP.C2b mainly consisted of type 2 and unclassified KIRP and was associated with *SETD2* mutation and DNA methylation cluster 1. Molecular subtype KIRP.C2c-CIMP was enriched in type 2 KIRP and associated with hypermethylation of the *CDKN2A* promoter and mutation of *FH (* 30). In the TCGA cohort, we compared the immune subtypes identified with the histological and molecular subtypes. In terms of histological subtypes, C3 was mainly composed of type 2 and C2 was mainly composed of type 1. The composition ratio of the histological subtypes was comparable among C1, C4, and C5 (**Figure 10A**). C3 was mainly presented in type 2 (**Figure 10B**). With regard to molecular subtypes, KIRP.C2a accounted for a large proportion of C3, whereas KIRP.C1 was dominant in C1, C2, C4, and C5 (**Figure 10C**). C3 was mainly presented in KIRP.C2a and KIRP.C2c-CIMP. C2 mainly appeared in KIRP.C1 and KIPR.C2b (**Figure 10D**).

### The Mutation Frequency of *TTN* Was Lower in the High Immunotherapy Response Group Than in the Low/Medium Immunotherapy Response Group

**Figure 11:**
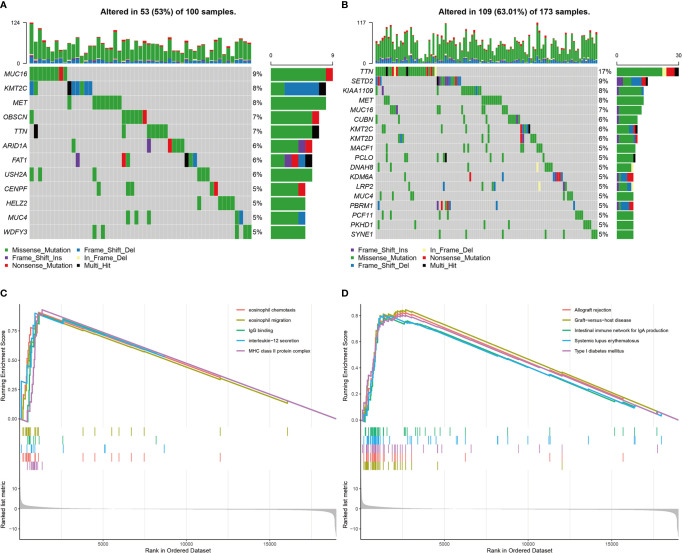
Landscapes of mutant genes in immunotherapy response groups and GSEA results. **(A)** The waterfall plot of genes with mutation frequency greater than 5% in the high immunotherapy response group. **(B)** The waterfall plot of genes with mutation frequency greater than 5% in the low/medium immunotherapy response group. **(C)** GO enrichments of GSEA between the high response group and low/medium group. **(D)** KEGG enrichments of GSEA between the high response group and low/medium group.

In the TCGA cohort, the genes with a mutation frequency greater than 5% were different in the high and low/medium immunotherapy response groups. *MUC16*, *KMT2C*, *MET*, *TTN*, and *MUC4* were common between the two groups. *OBSCN*, *ARID1A*, *FAT1*, *USH2A*, *CENPF*, *HELZ2*, and *WDFY3* were specific to the high immunotherapy response group. *SETD2*, *KIAA1109*, *CUBN*, *KMT2D*, *MACF1*, *PCLO*, *DNAH8*, *KDM6A*, *LRP2*, *PBRM1*, *PCF11*, *PKHD1*, and *SYNE1* were specific to the low/medium immunotherapy response group. The difference in the mutation frequency of *TTN* was most significant between these two groups. The high response group was 7% and the low/medium group was 17% (**Figures 11A, B**). GSEA performed on these two groups revealed that multiple immune-related functions and pathways such as eosinophil chemotaxis, eosinophil migration, IgG binding, interleukin-12 secretion, MHC class II protein complex, allograft rejection, graft-versus-host disease, the intestinal immune network for IgA production, systemic lupus erythematosus, and type I diabetes mellitus were significantly enriched in the high immunotherapy response group (**Figures 11C, D**).

## Discussion

Tumor immunotherapy targeting immune checkpoints has shown encouraging therapeutic effects. Because of the differences in individual responses of patients, screening of the beneficiary population for specific tumor subtypes and identifying new targets and biomarkers to develop single-therapy drugs and combined therapy programs still need to be intensively performed. Based on the computational biology method and the in-depth analysis of the multidimensional data of 289 patients with KIRP in the TCGA cohort and 103 patients with KIRP in the GEO cohort, our research shows the following. 1) There is a certain number of potentially functional immune cells in the tumors of patients with KIRP but the tumor-infiltrating CD8+ T cells are exhausted, causing them to fail to exert their antitumor effects. 2) The C1 and C3 immune subtypes that we identified may have the highest clinical benefit in reversing CD8+ T-cell exhaustion. Our comprehensive evaluation of the tumor immune microenvironment of patients with KIRP suggests that these patients may be suitable for immunotherapy that reverses CD8+ T-cell exhaustion.

**Figure 12:**
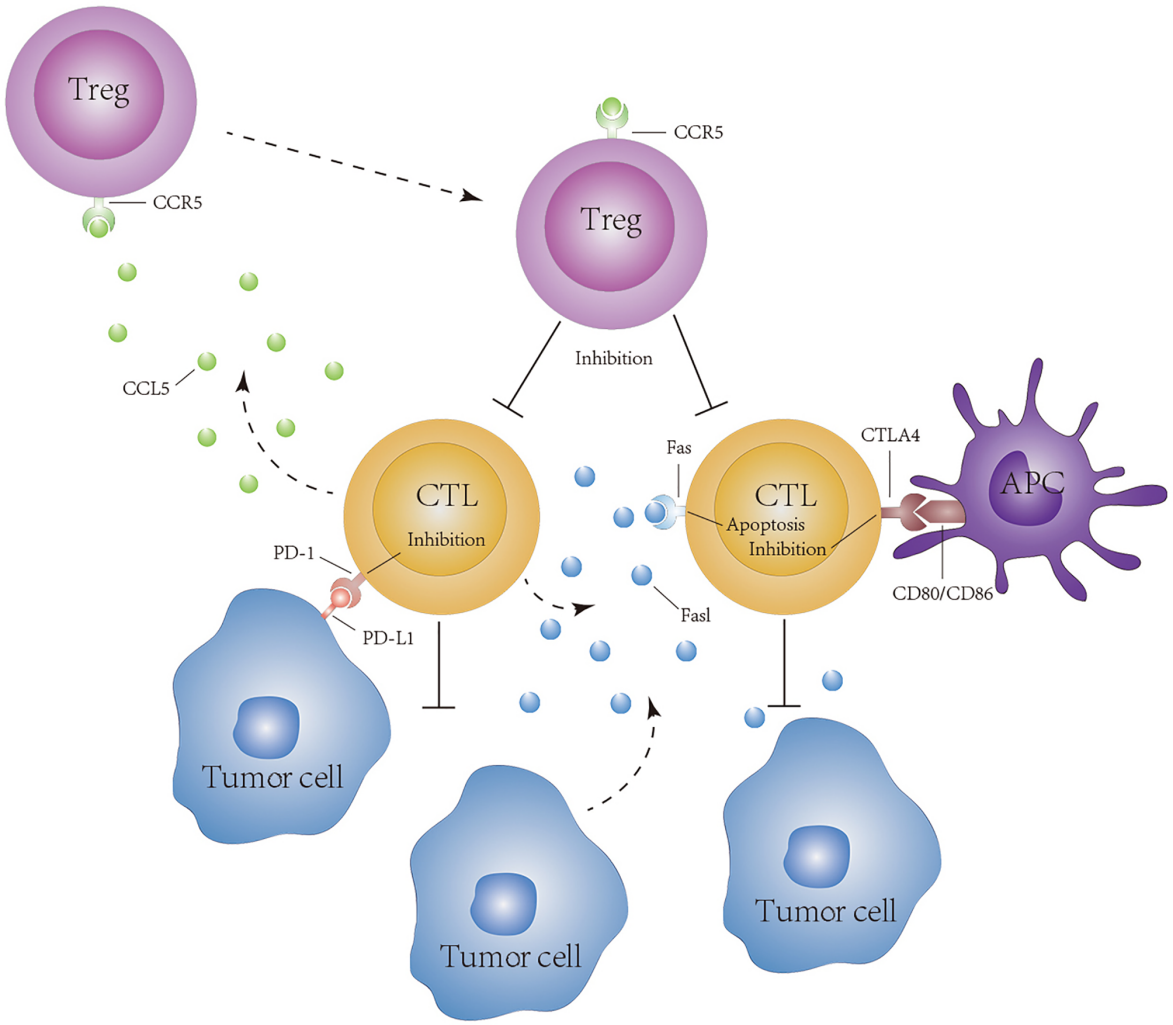
Hypothesis of CD8+ T cell exhaustion in tumors of KIRP patients. PD-1, CTLA4, CCL5, and FASLG reduce the cytotoxicity of lymphocytes to tumor cells.

Based on the comprehensive evaluation of the tumor immune microenvironment of patients with KIRP, we identified 12 hub genes that were closely related to this tumor microenvironment. These 12 hub genes were highly expressed in these patients and showed a strong correlation with CD8+ T cells. The roles of these 12 hub genes in tumors can be divided into two major groups: those with immunostimulatory functions (*CD2*, *CD8A*, *CD8B*, *CD3D*, *CD3E*, *EOMES*, and *GZMA*) and those with immunosuppressive functions (*PDCD1*, *CTLA4*, *FASLG*, and *CCL5*). Some of these genes are cancer suppressor genes. *CD2*, *CD8A*, *CD8B*, *CD3D*, and *CD3E* are membrane proteins on the surface of T cells and play an important role in the immune recognition and activation of T cells (31–33). *EOMES* has an important effect on the development of NK cells and the differentiation of CD8+ T cells (34, 35). *GZMA*, a cytotoxic protein secreted by NK cells and cytotoxic T cells, can induce caspase-dependent cell death (36). At present, the role of *NKG7* in cancer has not been elucidated. In addition, some of these genes are involved in the formation of an immunosuppressive microenvironment in the tumor. *CTLA4* is an inhibitory receptor found on the surface of T cells. It can downregulate the activity of T cells after binding to *CD80* and *CD86* on antigen-presenting cells (37, 38) (**Figure 12**). *PDCD1* belongs to the cell-surface immunoglobulin superfamily. After interacting with *PD-L1*, the proliferation and activity of tumor-specific T cells are inhibited (39) (**Figure 12**). *FASLG* is a member of the tumor necrosis factor superfamily. Soluble *FASLG* can kill effector immune cells and cause immune tolerance (40, 41) (**Figure 9**). In adoptive cell immunotherapy, when genetically engineered *Fas* receptor-negative T cells are used for cancer treatment, *Fas* receptor-negative T cells show longer durability and stronger antitumor immunity both in the periphery blood and in tumors of tumor-bearing animals (42). The chemokine *CCL5* can recruit Treg cells to enhance immune tolerance (43) (**Figure 12**). In our study, *CCL5* and Treg cells also showed a strong correlation (**Supplementary Figure 2**). Additionally, *CCL5* produced by immune cells can promote tumor growth and proliferation by regulating macrophage production of metalloproteinases or inducing epithelial–mesenchymal transformation in tumor cells (44, 45). There is evidence that drugs blocking the *CCR5*–*CCL5* axis or reducing *CCL5* production can be used in the clinical setting (46). It is worth noting that in addition to the common immune checkpoint targets such as *PD-1*, we found that *CCL5* and *FASLG* may play an important role in the formation of the KIRP tumor immunosuppressive microenvironment; however, studies on the role of *CCL5* and *FASLG* expression in KIRP have not been performed. Therefore, whether *CCL5* and *FASLG* and their receptors can be used as new targets for KIRP immunotherapy remains to be further studied.

The main finding of this study was to identify that patients with KIRP are suitable for receiving immunotherapy that can reverse CD8+ T-cell exhaustion. In our study, we found a strong co-expression relationship between some genes with immunosuppressive effects and those with immune-promoting effects in the purple module, indicating that the greater the activation potential and antitumor potential of CD8+ T cells, the greater the possibility of inhibition. In addition, our results showed that in the tumor of patients with KIRP, the numbers of Treg cells with immunosuppressive effects and CD8+ T cells continue to increase with tumor progression. This finding is consistent with the observation that the number of CD8+ T cells in RCC is positively correlated with the tumor grade but because of the immunosuppressive environment in renal cell carcinoma, TILs with lower proliferative activity cannot exert their function effectively (47, 48). Survival analysis showed that CD8+ T cells were significantly associated with poor prognosis of patients with KIRP. Based on the above findings, we can infer that KIRP belongs to the “hot” tumor category. Although there is a certain number of CD8+ T cells around KIRP tumor cells, because of an immunosuppressive environment and the expression of immunosuppressive receptors by CD8+ T cells, CD8+ T cells are exhausted, resulting in the inability of CD8+ T cells to exert their antitumor effects. Therefore, reversing exhausted CD8+ T cells and restoring their antitumor ability could be a reasonable choice in KIRP immunotherapy.

To find potentially suitable patients who could benefit the most from the treatment of reversing CD8+ T-cell exhaustion, we clustered a TCGA cohort of KIRP into five immune subtypes based on 12 hub genes associated with CD8+ T cells and validated these immune subtypes in the GEO cohort. C1 and C3 had the highest immune component, T-cell dysfunction score, and percentages of true responders to immunotherapy (C1: 81.7%, C3: 96.6%). C2 had the lowest immune component and T-cell dysfunction score. In terms of percentages of true esponders to immunotherapy, C2 had the lowest one (7.7%). C4 and C5 had moderate immune components, T-cell dysfunction scores, and percentages of true responders to immunotherapy (C4: 44.8%, C5: 29.5%). Therefore, among the five immune subtypes, high immunotherapy response subtypes C1 and C3 could obtain the best efficacy of reversing CD8+ T-cell exhaustion therapy. The low immunotherapy response subtype C2 was not suitable for reversing CD8+ T-cell exhaustion therapy. It is worth noting that although C1 and C3 are both subtypes of high immunotherapy response, the number of patients at higher tumor stages (III/IV) in C1 was 18.2% and that in C3 was 44.8%. This finding suggests that immunotherapeutic interventions for early-stage patients of type C1 may be effective in preventing patients from transitioning to late stages.

In conclusion, based on an in-depth analysis of the multiomic and multidimensional data of KIRP from the largest sample available currently, we found that patients with KIRP whose immune microenvironment exhibits “hot” tumor characteristics are suitable for receiving therapy that causes the reversal of CD8+ T-cell exhaustion, and their C1 and C3 immune subtypes may achieve the best therapeutic effect. Genes such as *CCL5* and *FASLG* may play a crucial role in the formation of the KIRP immunosuppressive microenvironment. Given the limited high-quality clinical data of KIRP, further experimental and clinical studies are required to confirm the above findings and explore the corresponding immunotherapy regimens.

## Data Availability Statement

Publicly available datasets were analyzed in this study. These data can be found here: https://cancergenome.nih.gov and https://www.ncbi.nlm.nih.gov/geo/, accession numbers GSE2748, GSE7023 and GSE26574.

## Author Contributions

BW: formal analysis, data curation, conceptualization, writing—original draft. MY: formal analysis, visualization. JHY: software, investigation. MJ: investigation. JA: investigation. JPY: investigation. JL: investigation. YKZ: software. YYZ: writing—review and editing, supervision, project administration, funding acquisition. All authors contributed to the article and approved the submitted version.

## Funding

This work was supported by the national natural science foundation of China (grants 81672523, 81472404, 81472403, 81272834, and 31000572); the 2018 support plan for innovative talents in colleges and universities of Liaoning province; the 2018 “million talents project” funded project of Liaoning province (grant 33013); and the 2019 Key R & D projects of Shenyang (grant 19-112-4-102). 

## Conflict of Interest

The authors declare that the research was conducted in the absence of any commercial or financial relationships that could be construed as a potential conflict of interest.

## Publisher’s Note

All claims expressed in this article are solely those of the authors and do not necessarily represent those of their affiliated organizations, or those of the publisher, the editors and the reviewers. Any product that may be evaluated in this article, or claim that may be made by its manufacturer, is not guaranteed or endorsed by the publisher.

## Abbreviations

TCGA, The Cancer Genome Atlas; KIRP, kidney renal papillary cell carcinoma; FASLG; Fas ligand; CCL5, C–C motif chemokine ligand 5; RCC, renal cell carcinoma; TIIC, tumor infiltrating immune cell; CTLA4, cytotoxic T-lymphocyte-associated protein 4; TIM-3, T-cell immunoglobulin mucin family member 3; PD-1, programmed cell death 1; ccRCC, clear cell renal cell carcinoma; GEO, Gene Expression Omnibus; WGCNA, weighted gene co-expression network analysis; ME, module eigengene; GS, gene significance; MS, module significance; PPI, protein–protein interaction; KM, Kaplan–Meier; ssGSEA, single-sample gene set enrichment analysis; TIDE, tumor immune dysfunction and exclusion; GSEA, gene set enrichment analysis; GO, Gene Ontology; KEGG, Kyoto Encyclopedia of Genes and Genomes; GZMA, Granzyme A; CD2, CD2 molecule; PDCD1, programmed cell death 1; CD8A, CD8A molecule; CD3D, CD3D molecule; EOMES, Eomesodermin; NKG7, natural killer cell granule protein 7; CD3E, CD3E molecule; CD8B, CD8B molecule; HPA, The Human Protein Atlas; LAG3, lymphocyte activating 3; PD-L1, programmed cell death 1 ligand 1; MET, tyrosine–protein kinase Met; SETD2, histone-lysine N-methyltransferase SETD2; CDKN2A, cyclin-dependent kinase inhibitor 2A; TTN, Titin; MUC16, Mucin 16; KMT2C, lysine methyltransferase 2C; MUC4, Mucin 4; OBSCN, obscurin; ARID1A, AT-rich interaction domain 1A; FAT1, FAT atypical cadherin 1; USH2A, Usher syndrome type IIa protein; CENPF, centromere protein F; HELZ2, helicase with zinc finger 2; WDFY3, WD repeat and FYVE domain-containing 3; SETD2, SET domain-containing 2; KIAA1109, transmembrane protein KIAA1109; CUBN, cubilin; KMT2D, lysine methyltransferase 2D; MACF1, microtubule actin cross-linking factor 1; PCLO, Piccolo presynaptic cytomatrix protein; DNAH8, dynein axonemal heavy chain 8; KDM6A, lysine demethylase 6A; LRP2, LDL receptor-related protein 2; PBRM1, polybromo 1; PCF11, PCF11 cleavage and polyadenylation factor subunit; PKHD1, polycystic kidney and hepatic disease 1 protein; SYNE1, spectrin repeat-containing nuclear envelope protein 1

## Glossary

**Table:**

TCGA	The Cancer Genome Atlas
KIRP	kidney renal papillary cell carcinoma
FASLG	Fas ligand
CCL5	C–C motif chemokine ligand 5
RCC	renal cell carcinoma
TIIC	tumor infiltrating immune cell
CTLA4	cytotoxic T-lymphocyte-associated protein 4
TIM-3	T-cell immunoglobulin mucin family member 3
PD-1	programmed cell death 1
ccRCC	clear cell renal cell carcinoma
GEO	Gene Expression Omnibus
WGCNA	weighted gene co-expression network analysis
ME	module eigengene
GS	gene significance
MS	module significance
PPI	protein–protein interaction
KM	Kaplan–Meier
ssGSEA	single-sample gene set enrichment analysis
TIDE	tumor immune dysfunction and exclusion
GSEA	gene set enrichment analysis
GO	Gene Ontology
KEGG	Kyoto Encyclopedia of Genes and Genomes
GZMA	Granzyme A
CD2	CD2 molecule
PDCD1	programmed cell death 1
CD8A	CD8A molecule
CD3D	CD3D molecule
EOMES	Eomesodermin
NKG7	natural killer cell granule protein 7
CD3E	CD3E molecule
CD8B	CD8B molecule
HPA	The Human Protein Atlas
LAG3	lymphocyte activating 3
PD-L1	programmed cell death 1 ligand 1
MET	tyrosine–protein kinase Met
SETD2	histone-lysine N-methyltransferase SETD2
CDKN2A	cyclin-dependent kinase inhibitor 2A
TTN	Titin
MUC16	Mucin 16
KMT2C	lysine methyltransferase 2C
MUC4	Mucin 4
OBSCN	obscurin
ARID1A	AT-rich interaction domain 1A
FAT1	FAT atypical cadherin 1
USH2A	Usher syndrome type IIa protein
CENPF	centromere protein F
HELZ2	helicase with zinc finger 2
WDFY3	WD repeat and FYVE domain-containing 3
SETD2	SET domain-containing 2
KIAA1109	transmembrane protein KIAA1109
CUBN	cubilin
KMT2D	lysine methyltransferase 2D
MACF1	microtubule actin cross-linking factor 1
PCLO	Piccolo presynaptic cytomatrix protein
DNAH8	dynein axonemal heavy chain 8
KDM6A	lysine demethylase 6A
LRP2	LDL receptor-related protein 2
PBRM1	polybromo 1
PCF11	PCF11 cleavage and polyadenylation factor subunit
PKHD1	polycystic kidney and hepatic disease 1 protein
SYNE1	spectrin repeat-containing nuclear envelope protein 1
