# Supplementary material for: Multidimensional Analyses of Tumor Immune Microenvironment Reveal the Possible Rationality of Immunotherapy and Identify High Immunotherapy Response Subtypes for Renal Papillary Cell Carcinoma
Source: Front Immunol. 2021 Aug 31;12:657951. doi: 10.3389/fimmu.2021.657951 (PMC8438207; doi:10.3389/fimmu.2021.657951)
Supplement: Supplementary file 1 [file DataSheet_1.zip › 657951/data description.pdf]

We converted all non-Excel format files such as TXT, maf and CSV format to Excel format. The green front is the file name. Due to the lack of clinical data for some samples of KIRP in TCGA, the comparison of clinical information between subtypes cannot be included in all subtype samples. Since the data set GSE7023 lacks PD-1 expression information, we only used the data sets GSE2748 and GSE26574 when comparing the expression level of PD-1 in subtypes.

Figure 1

Figure 1A: WGCNA algorithm was used based on files diffmRNAExp_TCGA.xlsx and CIBERSORT_filter_TCGA.xlsx, and figure 1A, supplementary figure1B, supplementary figure1C and supplementary figure1D were generated.
Figure 1B: File purple module_TCGA.xlsx.
Figure 1C and 1D: From online database Metascape(http://metascape.org/gp/index.html).

Figure 2

Figure 2A: File string_interactions_TCGA.xlsx.
Figure 2B: File WGCNA_interactions_TCGA.xlsx.
Figure 2C: Files purple module_TCGA.xlsx, string_MCC_TCGA.xlsx and WGCNA_MCC_TCGA.xlsx.
Figure 2D: hub gene expression in tumor_TCGA.xlsx.

Figure 3

Figure 3A: File hub gene expression_log2 transformed_TCGA.xlsx.
Figure 3B: From online database HPA(http://www.proteinatlas.org).
Figure 3C: File PCR.zip.
Figure 3D: From online database UALCAN((http://ualcan.path.uab.edu/analysis.html).

Figure 4

Figure 4A and 4B: From online database TIMER(https://cistrome.shinyapps.io/timer/).
Figure 4C and 4D: Files CIBERSORT_filter_TCGA.xlsx and clustering result_TCGA.xlsx.

Figure 5

Figure 5A, 5B and 5C: Consensus clustering was performed on file clustering file_TCGA.xlsx, and file clustering result_TCGA.xlsx was generated.

Figure 5D: File survival analysis_TCGA.xlsx.
Figure 5E: Files TIMER result_TCGA.xlsx and clustering result_TCGA.xlsx.
Figure 5F: Files stage_TCGA.xlsx and clustering result_TCGA.xlsx.

Figure 6

Figure 6A: Files ssgseaOut_TCGA.xlsx and clustering result_TCGA.xlsx.
Figure 6B: Files scores_TCGA.xlsx and clustering result_TCGA.xlsx.

Figure 7

Figure 7A and 7B: Files TIDE_TCGA.xlsx and clustering result_TCGA.xlsx.
Figure 7C: File immune checkpoint_TCGA.xlsx and clustering result_TCGA.xlsx.

Figure 8

Figure 8A, 8B and 8C: Consensus clustering was performed on file clustering file_GEO.xlsx, and file clustering result_GEO.xlsx was generated.
Figure 8D: Files TIMER_GEO.xlsx and clustering result_GEO.xlsx.

Figure 9

Figure 9A: Files ssgseaOut_GEO.xlsx and clustering result_GEO.xlsx.
Figure 9B: Files scores_GEO.xlsx and clustering result_GEO.xlsx.
Figure 9C: Files immune checkpoint_GSE2748_GSE7023_GSE26574.xlsx, PD-1_GSE2748_GSE26574.xlsx and clustering result_GEO.xlsx.

Figure10

Figure 10A and 10B: File immune_subtypes and histological_subtypes_TCGA.xlsx.
Figure 10C and 10D: File immune_subtypes and molecular_subtypes_TCGA.xlsx.

Figure 11

Figure 11A: File mutation file of high response group_TCGA.xlsx.
Figure 11B: File mutation file of low and medium response group_TCGA.xlsx.
Figure 11C and 11D: File GSEA_file_TCGA.xlsx.

Supplementary figure 1

Supplementary figure 1A: Files up.xlsx and down.xlsx.

Supplementary figure 1B, 1C and 1D: File diffmRNAExp_TCGA.xlsx.
Supplementary figure 1E: File CIBERSORT_filter_TCGA.xlsx.

Supplementary figure 2

From online database TISIDB(http://cis.hku.hk/TISIDB/index.php)

Supplementary Table 1 and Supplementary Table 2
From online database Metascape(http://metascape.org/gp/index.html)
