# Supplementary material for: Multidimensional Analyses of Tumor Immune Microenvironment Reveal the Possible Rationality of Immunotherapy and Identify High Immunotherapy Response Subtypes for Renal Papillary Cell Carcinoma
Source: Front Immunol. 2021 Aug 31;12:657951. doi: 10.3389/fimmu.2021.657951 (PMC8438207; doi:10.3389/fimmu.2021.657951)
Supplement: Supplementary file 2 [file DataSheet_2.docx]

Supplementary figures


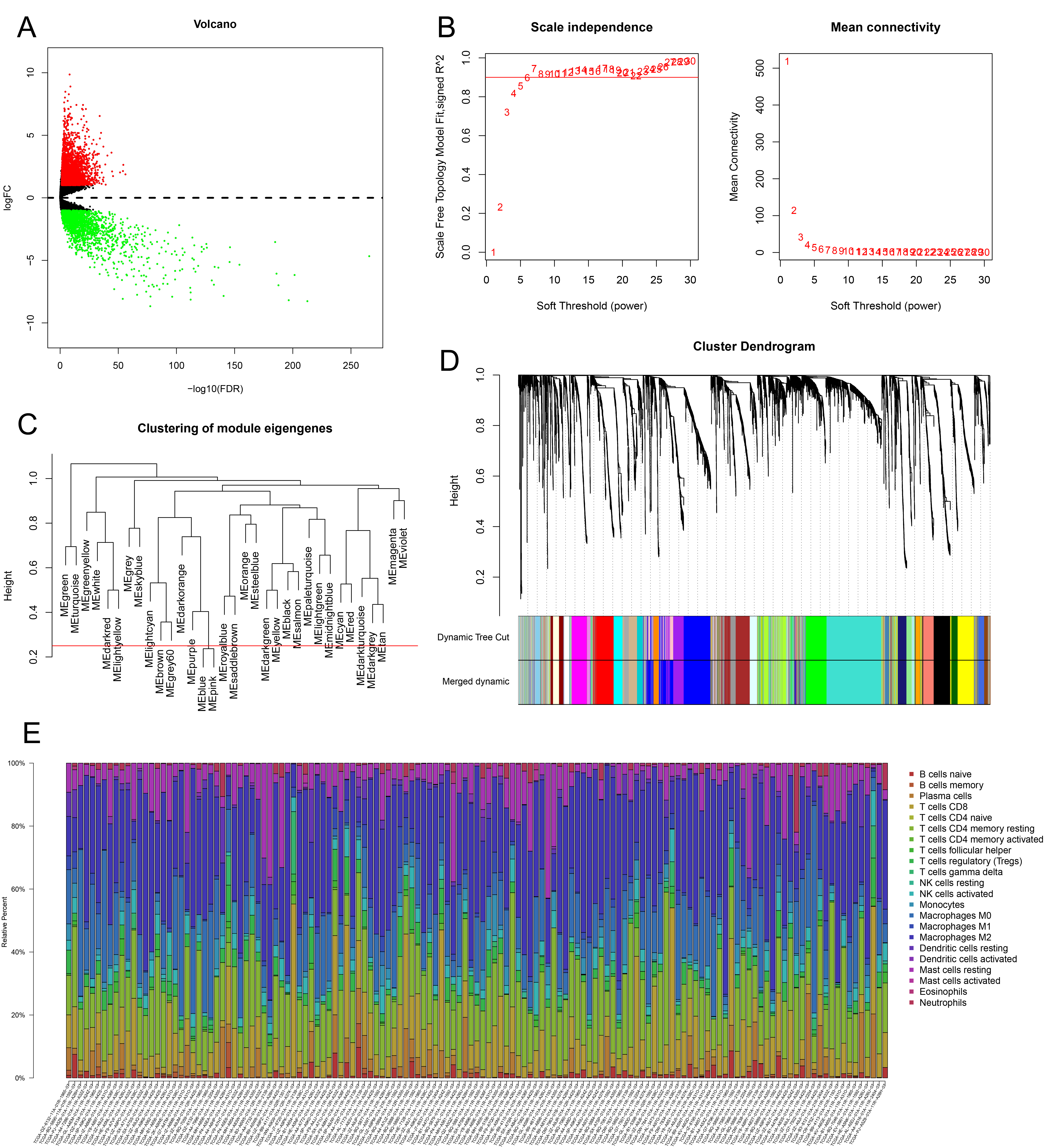


**Supplementary figure 1.** Construction of a co-expression network of KIRP differential genes and evaluation of the fraction of immune cells:(**A**) The X axis represents the -log10 conversion of FDR, and the Y axis represents the log2 conversion of fold-changes. The red dot is the up-regulated differential gene, and the green dot is the down-regulated differential gene. (**B**) Use soft threshold power analysis to obtain the scale-free fitting index of the network topology. (**C, D**) Perform hierarchical clustering analysis and merge similar modules, each color represents a module in the co-expression network constructed by WGCNA. (**E**) The histogram shows the relative fraction of 22 immune cell subtypes in each sample.


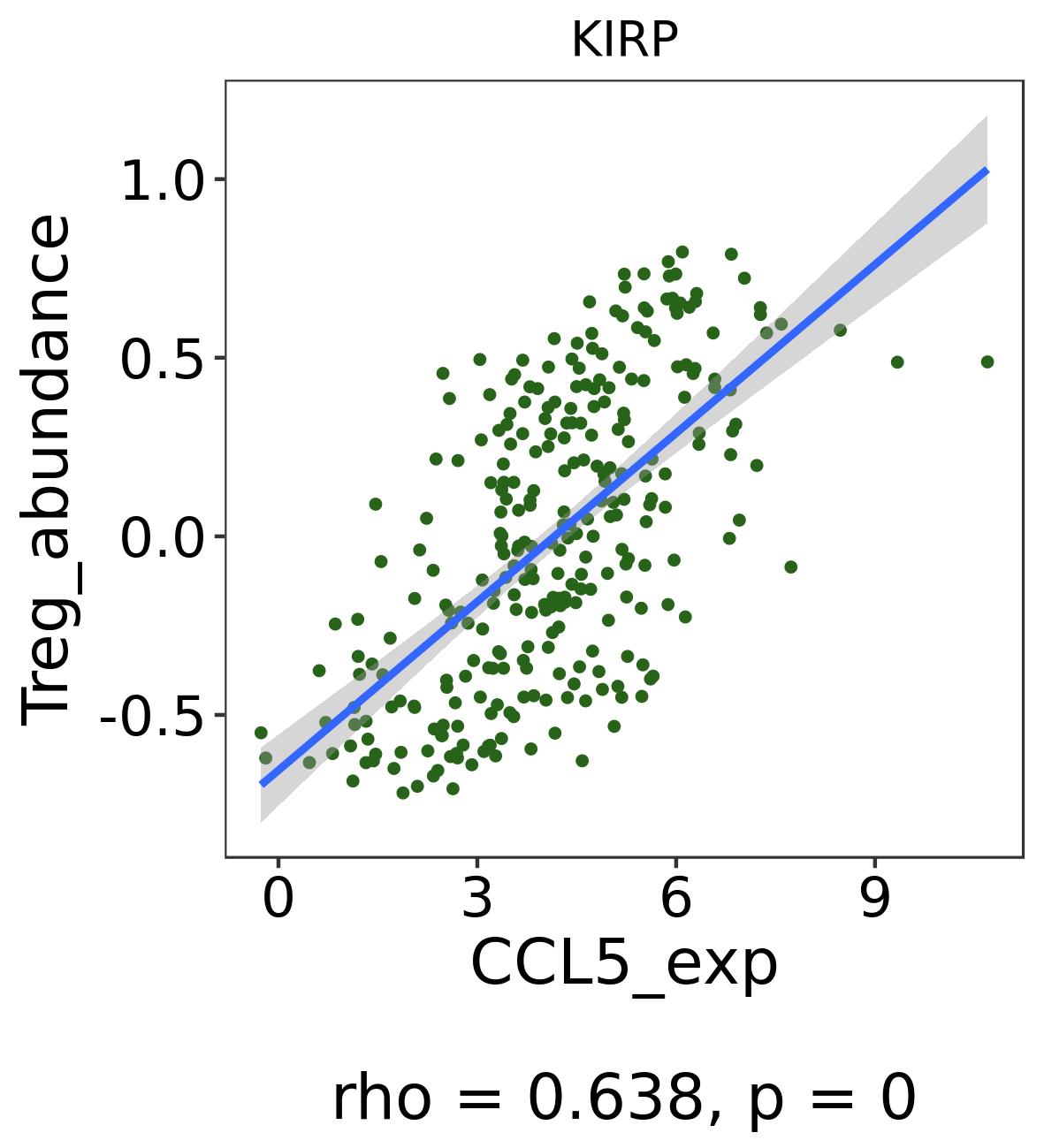


**Supplementary figure 2.** Scatter diagram of CCL5 and Treg cells.
