# Supplementary material for: Multidimensional Analyses of Tumor Immune Microenvironment Reveal the Possible Rationality of Immunotherapy and Identify High Immunotherapy Response Subtypes for Renal Papillary Cell Carcinoma
Source: Front Immunol. 2021 Aug 31;12:657951. doi: 10.3389/fimmu.2021.657951 (PMC8438207; doi:10.3389/fimmu.2021.657951)
Supplement: Supplementary file 3 [file Table_1.doc]

**Supplementary Table 1. Detail results of GO and KEGG enrichment analysis of the purple model**

| **GO** | **Category** | **Description** | **Count** | **%** | **Log10(P)** | **Log10(q)** |
| --- | --- | --- | --- | --- | --- | --- |
| GO:0042110 | GO Biological Processes | T cell activation | 36 | 21.43 | -26.47 | -22.15 |
| M54 | Canonical Pathways | PID IL12 2PATHWAY | 15 | 8.93 | -18.74 | -15.02 |
| GO:0034341 | GO Biological Processes | response to interferon-gamma | 20 | 11.90 | -16.90 | -13.43 |
| GO:0019221 | GO Biological Processes | cytokine-mediated signaling pathway | 33 | 19.64 | -16.10 | -12.86 |
| GO:0002250 | GO Biological Processes | adaptive immune response | 31 | 18.45 | -15.97 | -12.76 |
| GO:0002228 | GO Biological Processes | natural killer cell mediated immunity | 11 | 6.55 | -12.03 | -9.11 |
| hsa04612 | KEGG Pathway | Antigen processing and presentation | 11 | 6.55 | -11.27 | -8.38 |
| hsa04060 | KEGG Pathway | Cytokine-cytokine receptor interaction | 17 | 10.12 | -11.19 | -8.34 |
| GO:0002695 | GO Biological Processes | negative regulation of leukocyte activation | 13 | 7.74 | -9.43 | -6.78 |
| hsa04650 | KEGG Pathway | Natural killer cell mediated cytotoxicity | 11 | 6.55 | -8.66 | -6.14 |
| GO:0045088 | GO Biological Processes | regulation of innate immune response | 18 | 10.71 | -8.60 | -6.11 |
| GO:0032609 | GO Biological Processes | interferon-gamma production | 10 | 5.95 | -8.20 | -5.77 |
| GO:0009615 | GO Biological Processes | response to virus | 15 | 8.93 | -7.92 | -5.53 |
| GO:0001817 | GO Biological Processes | regulation of cytokine production | 22 | 13.10 | -7.89 | -5.51 |
| GO:0030101 | GO Biological Processes | natural killer cell activation | 8 | 4.76 | -6.92 | -4.64 |
| GO:0002507 | GO Biological Processes | tolerance induction | 5 | 2.98 | -6.16 | -3.97 |
| GO:0036037 | GO Biological Processes | CD8-positive, alpha-beta T cell activation | 5 | 2.98 | -6.07 | -3.88 |
| GO:0032496 | GO Biological Processes | response to lipopolysaccharide | 12 | 7.14 | -5.37 | -3.25 |
| GO:0070231 | GO Biological Processes | T cell apoptotic process | 5 | 2.98 | -4.70 | -2.67 |
| GO:0042832 | GO Biological Processes | defense response to protozoan | 4 | 2.38 | -4.54 | -2.53 |
