# Supplementary material for: Multidimensional Analyses of Tumor Immune Microenvironment Reveal the Possible Rationality of Immunotherapy and Identify High Immunotherapy Response Subtypes for Renal Papillary Cell Carcinoma
Source: Front Immunol. 2021 Aug 31;12:657951. doi: 10.3389/fimmu.2021.657951 (PMC8438207; doi:10.3389/fimmu.2021.657951)
Supplement: Supplementary file 4 [file Table_2.doc]

**Supplementary Table 2. The functional analysis of six closely-linked network components from the purple model**

| **Color** | **MCODE** | **GO** | **Description** | **Log10(P)** |
| --- | --- | --- | --- | --- |
| 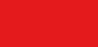 | MCODE_1 | R-HSA-375276 | Peptide ligand-binding receptors | -12.5 |
| 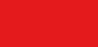 | MCODE_1 | R-HSA-380108 | Chemokine receptors bind chemokines | -12.4 |
| ­­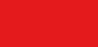 | MCODE_1 | GO:0070098 | chemokine-mediated signaling pathway | -11.5 |
| 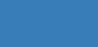 | MCODE_2 | R-HSA-389948 | PD-1 signaling | -12.2 |
| 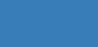 | MCODE_2 | R-HSA-388841 | Costimulation by the CD28 family | -10.2 |
| 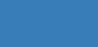 | MCODE_2 | hsa04660 | T cell receptor signaling pathway | -9.5 |
| 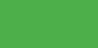 | MCODE_3 | hsa03050 | Proteasome | -11.0 |
| 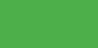 | MCODE_3 | GO:0010499 | proteasomal ubiquitin-independent protein catabolic process | -8.6 |
| 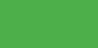 | MCODE_3 | R-HSA-8878171 | Transcriptional regulation by RUNX1 | -8.0 |
| 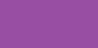 | MCODE_4 | hsa04612 | Antigen processing and presentation | -10.0 |
| 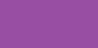 | MCODE_4 | R-HSA-198933 | Immunoregulatory interactions between a Lymphoid and a non-Lymphoid cell | -9.1 |
| 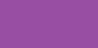 | MCODE_4 | hsa04514 | Cell adhesion molecules (CAMs) | -8.9 |
| 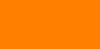 | MCODE_5 | R-HSA-375276 | Peptide ligand-binding receptors | -8.3 |
| 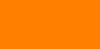 | MCODE_5 | R-HSA-416476 | G alpha (q) signalling events | -8.2 |
| 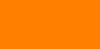 | MCODE_5 | R-HSA-373076 | Class A/1 (Rhodopsin-like receptors) | -7.5 |
| 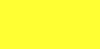 | MCODE_6 | R-HSA-202430 | Translocation of ZAP-70 to Immunological synapse | -9.4 |
| 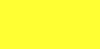 | MCODE_6 | R-HSA-202403 | TCR signaling | -6.9 |
| 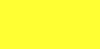 | MCODE_6 | GO:0050852 | T cell receptor signaling pathway | -6.3 |
